# Association between prehospital fluid resuscitation with crystalloids and outcome of trauma patients in Asia by a cross-national multicenter cohort study

**DOI:** 10.1038/s41598-022-06933-x

**Published:** 2022-03-08

**Authors:** Chih-Wei Sung, Jen-Tang Sun, Edward Pei-Chuan Huang, Sang Do Shin, Kyoung Jun Song, Ki Jeong Hong, Sabariah Faizah Jamaluddin, Do Ngoc Son, Ming-Ju Hsieh, Matthew Huei-Ming Ma, Li-Min Hsu, Wen-Chu Chiang, The PATOS Clinical Research Network, Ramana Rao, George P. Abraham, T. V. Ramakrishnan, Sabariah Faiwah Jamaluddin, Mohd Amin Bin Mohidin, Al-Hilmi Saim, Lim Chee Kean, Cecilia Anthonysamy, Shah Jahan Din Mohd Yssof, Kang Wen Ji, Cheah Phee Kheng, Shamila Bt Mohamad Ali, Periyanayaki Ramanathan, Chia Boon Yang, Hon Woei Chia, Hafidahwati Binti Hamad, Samsu Ambia Ismail, Wan Rasydan B. Wan Abdullah, Hideharu Tanaka, Akio Kimura, Bernadett Velasco, Carlos D. Gundran, Pauline Convocar, Nerissa G. Sabarre, Patrick Joseph Tiglao, Ki Jeong Hong, Kyoung Jun Song, Joo Jeong, Sung Woo Moon, Joo-yeong Kim, Won Chul Cha, Seung Chul Lee, Jae Yun Ahn, Kang Hyeon Lee, Seok Ran Yeom, Hyeon Ho Ryu, Su Jin Kim, Sang Chul Kim, Ray-Heng Hu, Jen Tang Sun, Ruei-Fang Wang, Shang-Lin Hsieh, Wei-Fong Kao, Sattha Riyapan, Parinya Tianwibool, Phudit Buaprasert, Osaree Akaraborworn, Omer Ahmed Al Sakaf, Saleh Fares, Le Bao Huy, Do Ngoc Son, Nguyen Van Dai

**Affiliations:** 1 Department of Emergency Medicine, National Taiwan University Hospital Hsin-Chu Branch, Hsinchu City, Taiwan; 2 Department of Emergency Medicine, Far Eastern Memorial Hospital, New Taipei City, Taiwan; 3 Department of Emergency Medicine, National Taiwan University Hospital, Taipei City, Taiwan; 4 SMG-SNU Boramae Medical Center, Seoul, Korea; 5 Faculty of Medicine, Universiti Teknologi MARA, Shah Alam, Malaysia; 6 Center for Critical Care Medicine, Bach Mai Hospital, Hanoi City, Vietnam; 7 Department of Emergency Medicine, National Taiwan University Hospital, Yun-Lin Branch, Douliu City, Taiwan; 8 Department of Traumatology and Critical Care, National Taiwan University Hospital, Taipei City, Taiwan; 9 GVK EMRI, Hyderabad, India; 10 Indian Institute of Emergency Medical Services, Chennai, India; 11 Sri Ramachandra Medical Center, Chennai, India; 12 Sungai Buloh Hospital, Petaling District, Malaysia; 13 Sultanah Aminah Hospital, Johor Bahru, Malaysia; 14 Seri Manjung Hospital, Seri Manjung, Malaysia; 15 Pulau Pinang Hospital, George Town, Malaysia; 16 Serdang Hospital, Sepang, Malaysia; 17 Kuala Lumpur Hospital, Kuala Lumpur, Malaysia; 18 Sarikei Hospital, Sarikei, Malaysia; 19 Sabah Women and Children’s Hospital, Kota Kinabalu, Malaysia; 20 Ampang Hospital, Ampang Jaya, Malaysia; 21 Kajang Hospital, Kajang, Malaysia; 22 Miri Hospital, Miri, Malaysia; 23 Sarawak General Hospital, Kuching, Malaysia; 24 Queen Elizabeth II Hospital, Kota Kinabalu, Malaysia; 25 Teluk Intan Hospital, Teluk Intan, Malaysia; 26 Raja Perempuan Zainab II Hospital, Kota Bharu, Malaysia; 27 Kokushikan University, Tokyo, Japan; 28 National Center for Global Health and Medicine Hospital, Tokyo, Japan; 29 East Avenue Medical Center, Quezon City, Philippines; 30 Philippine College of Emergency Medicine, Parañaque, Philippines; 31 Southern Philippines Medical Centre, Davao City, Philippines; 32 Pasig City General Hospital, Pasig, Philippines; 33 Corazon Locsin Montelibano Memorial Regional Hospital, Bacolod, Philippines; 34 Boramae Medical Center, Seoul, South Korea; 35 Seoul National Univerisity Bundang Hospital, Seoul, South Korea; 36 Korea University Ansan Hospital, Ansan-si South, Korea; 37 Samsung Medical Center, Seoul, South Korea; 38 Dongguk University Ilsan Hospital, Goyang-si, South Korea; 39 Kyungpook National University Hospital, Daegu, South Korea; 40 Wonju Severance Christian Hospital, Wonju, South Korea; 41 Pusan National University Hospital, Busan, South Korea; 42 Chonnam National University Hospital, Gwangju, South Korea; 43 Korea University Anam Hospital, Seoul, South Korea; 44 Chungbuk National University Hospital, Cheongju-si, South Korea; 45 Shin Kong Wu Ho-Su Memorial Hospital, Taipei City, Taiwan; 46 Mackay Memorial Hospital, Taipei City, Taiwan; 47 Taipei City Hospital, Taipei City, Taiwan; 48 Faculty of Medicine Siriraj Hospital, Bangkok, Thailand; 49 Faculty of Medicine, Chiangmai University, Chiang Mai, Thailand; 50 Faculty of Medicine Vajira Hospital, Navamindradhiraj University, Bangkok, Thailand; 51 Prince of Songkla University, Songkhla, Thailand; 52 Dubai Corporation for Ambulance Services, Dubai, United Arab Emirates; 53 National Ambulance LLC, Abu Dhabi, United Arab Emirates; 54 Thong Nhat Hospital, Ho Chi Minh City, Vietnam; 55 Viet Tiep Hospital, Haiphong, Vietnam; 56 Department of Emergency and Critical Care Medicine, Hanoi Medical University, Hanoi, Vietnam; 57 Faculty of Medicine, University of Medicine and Pharmacy, Vietnam National University, Hanoi, Vietnam

**Keywords:** Medical research, Risk factors

## Abstract

Prehospital fluid resuscitation with crystalloids in patients following trauma remain controversial. This study aimed to investigate the association between prehospital fluid resuscitation and outcomes of trauma patients in Asia. We conducted a retrospective cohort study of trauma patients between 2016 and 2018 using data from the Pan-Asia Trauma Outcomes Study (PATOS) database. Prehospital fluid resuscitation was defined as any administration of intravenous crystalloid fluid on the ambulance before arrival to hospitals. The outcomes were in-hospital mortality and poor functional outcomes, defined as Modified Rankin Scale ≥ 4. Propensity score matching (PSM) was used to equalize potential prognostic factors in both groups. This study included 31,735 patients from six countries in Asia, and 4318 (13.6%) patients had ever received prehospital fluid resuscitation. The patients receiving prehospital fluid resuscitation had a higher risk of in-hospital mortality, with an adjusted odds ratio (aOR) of 2.02, 95% confidence interval (CI) 1.32–3.10, *p* = 0.001 in PSM analysis. Prehospital fluid resuscitation was also associated with poor functional outcomes, with an OR 1.73, 95% CI: 1.48–2.03, *p* < 0.001 in PSM analysis. Prehospital fluid resuscitation in patients with major trauma (injury severity score ≥ 16) presented a higher risk of poor functional outcomes (aOR = 2.65, 95% CI: 1.89–3.73 in PSM analysis, p_*interaction*_ = 0.006) via subgroup analysis. Prehospital fluid resuscitation of trauma patients is associated with higher in-hospital mortality and poor functional outcomes in the subgroup in countries studied.

## Introduction

Traumatic injury is a universal problem that causes unexpected consequences and extensive medical and social cost burden for years. In the United States, there are 136,000 trauma-related deaths annually^1^. Trauma with hemorrhagic events accounted for 30% to 40% of deaths within the first 24 h^2^. The Advanced Trauma Life Support (ATLS) guidelines published by the American College of Surgeons recommends that the evaluation of a patient’s circulation status is important after a stabilized state of airway and breath. Administration of intravenous fluids in patients with obvious hemorrhage or systolic blood pressure below 90 mmHg may improve outcomes^3^. Fluid resuscitation is helpful in replacing insufficient body volume to maintain minimal perfusion of vital organs before a definitive therapy is achieved, and it has been a component of trauma care in the prehospital setting by emergency medical services (EMS) since the 1970s.

Whether prehospital fluid resuscitation in traumatic patients can reduce mortality or affect functional outcome remains controversial. Haut et al. conducted a retrospective cohort study of patients from the American College of Surgeons National Trauma Data Bank (NTDB). The authors discouraged the routine use of prehospital fluid resuscitation for all trauma patients. However, the database did not report prehospital transport times or differentiate urban versus rural care; consequently, confounders during transportation could not be identified^4^. Other observational cohort studies also suggested that aggressive or excessive prehospital fluid resuscitation might be detrimental to patients with trauma^5–7^. However, insufficient information such as post-transfusion complications, coagulation factors, and unmatched cases and controls limited the explanation. Years later, the concept of a customized or personalized strategy for prehospital fluid resuscitation developed. Geeraedts et al. considered a personalized fluid transfusion protocol for traumatic patients. However, they did not adjust for types of resuscitation fluids, including crystalloids, colloids, and blood products^8^. The Cochrane review summary in 2014 concluded that there was no evidence for or against prehospital fluid resuscitation in patients with traumatic hemorrhage^9^.

Trauma patients generally include a wide range of age, gender, and ethnicity. There has never been a study to investigate the association between prehospital crystalloid fluid resuscitation and outcomes across countries in Asians. Public health policies differ between countries and the protocol may vary between EMS systems and hospital registries. Whether prehospital fluid resuscitation is beneficial in traumatic patients remains controversial^9, 10^. Our study aimed to investigate the association between prehospital fluid resuscitation and outcomes in patients with trauma in Asian countries.

## Methods

### Study design and setting

This cross-national, multicenter, retrospective cohort study was conducted by reviewing the patients’ data from the Pan-Asia Trauma Outcomes Study (PATOS), which was proposed in 2013 and initiated in November 2015. The PATOS database was developed to build an international and multicenter registry of traumatic injury patients across the Asia–Pacific countries. The large sample size and multinational nature of this registry provide an analysis of epidemiologic data, identification of risk factors, evaluation of interventions, and provide evidence for optimal management in trauma care^11, 12^. The subjects were enrolled in the PATOS research network, which collects traumatic patients transported by EMS providers between January 1, 2016 and November 31, 2018.

The PATOS coordination center provides details on the registry, and the principal investigator in each site is responsible for collecting the retrospective data. Some of the participating hospitals recorded the data directly, while others transcribed the data. The participating centers were tertiary hospitals in which the quality of care in patients who suffered traumatic injury was high in their respective countries, even though they did not share common guidelines. Different participating hospitals had different numbers of patients. Korea and Malaysia are the top two contributing countries in the PATOS. The variables were coded locally. The PATOS coordination center monthly quality conference is responsible for the management of missing data and obvious outliers. Any inconsistency between the participating hospitals was decided at the monthly conference before the COVID-19 pandemic. The current collected cohort partially represents all patients who suffered a traumatic injury and aims to cover all the populations as the participating centers increase^12^. The current collected cohort is partially representative of all trauma patients, and this cohort is toward covering all population when the participating centers increase. This study is reported as per the Strengthening the Reporting of Observatinal Studies in Epidemiology (STROBE) guideline (Supplementary Table S1)^13^.

### Ethics declarations and approval for human experiments

The PATOS collaboration was approved by the institutional review board of the Far Eastern Memory Hospital and National Taiwan University Hospital. The informed consent was waived because the data distributed by the PATOS coordination canter were fully anonymized and de-identification at the time they were accessed by the authors. All methods were performed in accordance with the relevant guidelines and regulations.

### Selection of participants and interventions

We included patients aged > 18 years. The patients suffered traumatic injury and were brought to the hospital by ambulance. The hospitals to which the patients were transported were eligible for providing traumatic care. Prehospital fluid resuscitation in traumatic patients was defined as any crystalloid fluids administered intravenously in the field or on the ambulance during the prehospital phase. The emergency medical technician examined the patients. In many EMS regions, when a patient suffered from potential fluid loss, such as bleeding, hypotension, tachycardia, and prolonged capillary refill time, EMT would initiate the prehospital fluid resuscitation. The patients who received prehospital fluid resuscitation were classified into the fluid group, while those who did not receive any fluid were categorized into the non-fluid group.

### Measurements

We included the variables or factors that originated from four different categories: injury epidemiologic factors, EMS factors, emergency department care factors, and hospital care factors. For injury epidemiology, variables including age, sex, country or citizenship, research institute, mechanism of injury, location of injury, and type of injury were collected. The mechanism, location, and type of injury were recorded by EMS providers and confirmed by physicians. EMS factors included date and time of ambulance to the accident site, duration of patient management at scene, date and time of ambulance arrival at hospital, fluid transfusion amount and type, and prehospital vital signs. At ED management, blood transfusion was recorded as a potential factor. Vital signs were collected at the ED triage. Outcome variables were abstracted from inpatient medical records. The variables of interest were abstracted in the predefined data collection form for further analysis.

### Outcomes

The primary outcome was in-hospital mortality. The secondary outcome was poor functional outcome assessed by the physicians or nurse staff at the time of hospital discharge. Poor functional outcomes were measured using the modified Rankin scale (MRS). . MRS has been regarded as an index for measuring the degree of disability in patients with brain injury, stroke, and neurosurgery. The quality of life is important after recovery for patients who have suffered a traumatic injury. Functional outcome, measured by MRS, is regarded as an index that reflects functional recovery after trauma^11, 14^. Compared to the Cerebral Performance Category, another standard used to assess neurologic outcomes following cardiac arrest, MRS mainly focusses on functional domains and can be easily determined using a chart review^15^.

MRS is a 6-level scale where no significant disability, slight disability, or moderate disability (MRS 0–3) is defined as favorable functional outcomes, while moderately severe disability, severe disability, and death (MRS 4–6) were categorized as poor functional outcomes^16^.

### Statistical analyses

Data collection and processing were performed by the PATOS coordination center. The data were collected from three components: prehospital data, EMS dispatch information, and medical records in the hospital. Prehospital data were recorded from ambulance run sheets. EMS information is collected by dispatch records. The treatment, diagnosis, and outcomes of each patient were recorded from medical records in EDs, intensive care units, and wards. Personal identifiable data were preliminarily used for linking EMS agencies and hospitals. Once linked, all variables of interest were permanently de-identified. Data were cleaned and managed by the Data Management Team of the participating institution to address and correct significant errors. Any data available for individual investigators from the PATOS Database were in de-identified format.

A Shapiro–Wilk test was performed to test for the normality of the variables^17^. For continuous variables with normal distribution, the data were presented as mean ± standard deviation and compared using an independent sample t-test. For variables without normal distribution, the median with lower and upper quartiles were presented. Dichotomous and categorical data were presented as numbers (percentages) and were compared using a chi-squared test. To avoid potential unbalanced demographics that cause selection biases between the fluid and non-fluid resuscitation groups, propensity score matching (PSM) was conducted to balance the distribution of the characteristics. The propensity score was the predicted probability receiving fluid resuscitation, given the values of covariates, using a multivariable logistic regression analysis without considering interaction effects among covariates. Each patient in the fluid resuscitation group was matched with one counterpart in the non-fluid resuscitation group by factors including age, sex, country, mechanism and location of injury, diagnosis, Injury Severity Score (ISS), shock, and vital signs. To calculate ISS, take the highest AIS severity code in each of the three most severely injured ISS body parts, square each AIS code and add the three squared numbers for an ISS. The matching was processed using a greedy nearest neighbor algorithm with a caliper of 0.2 times the standard deviation of the logit of the propensity score, with random matching order and without replacement. The quality of matching was checked using the *p*-value (*p* < 0.05) between the groups, where a value of greater than 0.1 was considered a negligible difference.

The risk of in-hospital mortality and poor functional outcome between the fluid and non-fluid resuscitation groups in the PSM cohort was compared using a univariate logistic regression analysis, in which the outcome dependency of patients among the same matched pair was accounted for using the robust standard error and working correlation matrix of the generalized estimating equation. A further subgroup analysis of the PSM cohort was performed to evaluate the effect of fluid resuscitation across different levels of subgroup variables, including sex, age, shock at ambulatory, major trauma (ISS ≥ 16), and mechanism of injury. Multivariable logistic regression using the original cohort adjusted for the same covariates in the PSM model was also performed.

Due to a lot of patients were excluded in the propensity score matched cohort, we conducted inverse probability of treatment weighting (IPTW) with stabilized weight based on propensity score to increase the statistical power. To reduce the impact of extreme weight, the weights were truncated at the 97th percentile. A two-sided *P* value of < 0.05 was considered statistically significant, and no adjustment of multiple testing (multiplicity) was performed in this study. All statistical analyses were performed using SAS version 9.4 (SAS Institute, Cary, NC).

## Results

### Characteristics of study subjects

**Figure 1:**
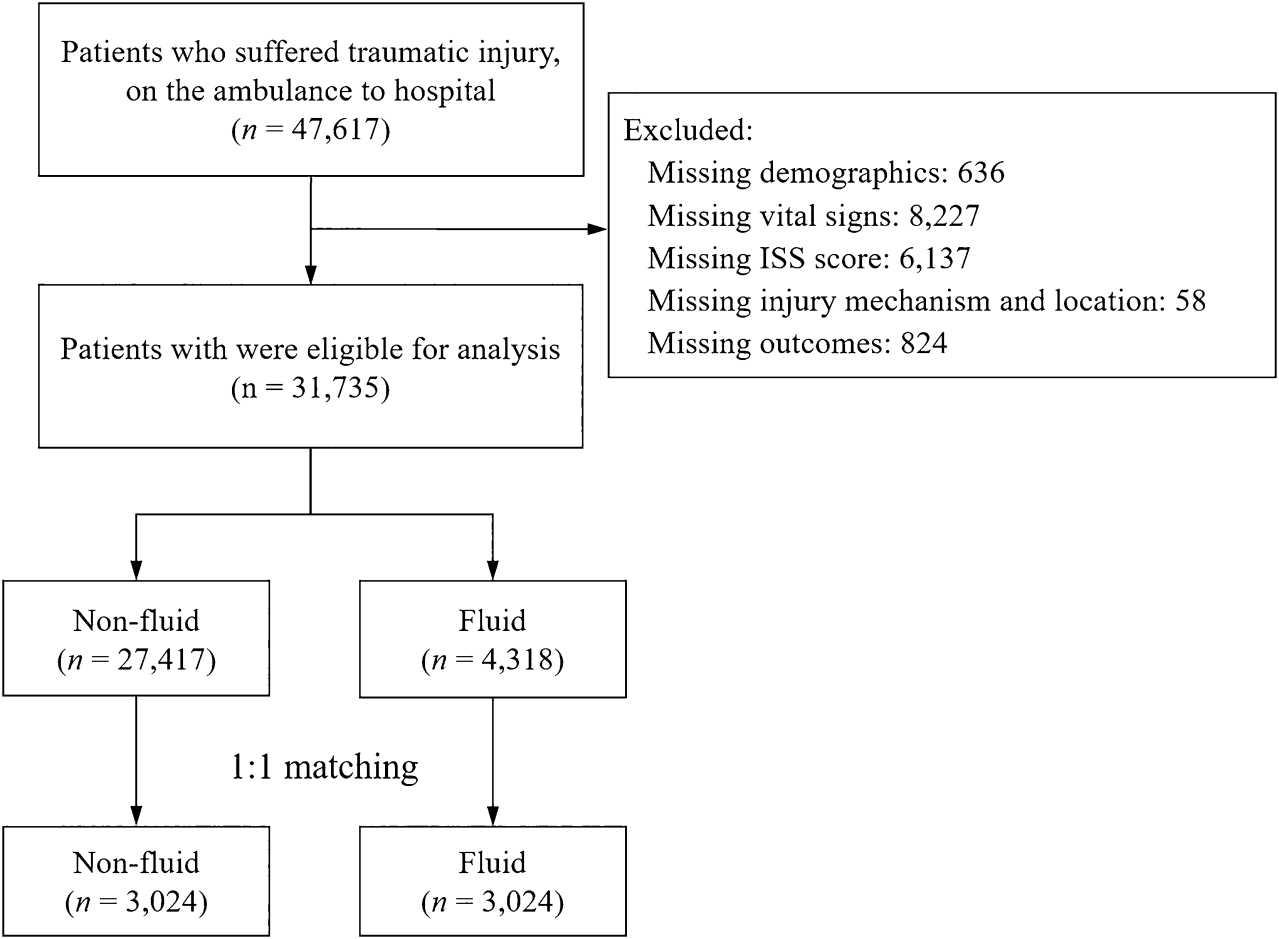
Flow diagram of the enrolled patients. Figures are made by Microsoft PowerPoint 2019 and SPSS version 26.

We preliminary enrolled 47,617 trauma patients who received crystalloid fluid resuscitation on arrival to hospitals from the PATOS registry. After reviewing the database, 636, 8227, 6137, 58, and 824 patients were excluded because there were no basic demographics, absent or extremely over-standard values in vitals, blanks for ISS scores, absent mechanisms of injury, and loss of follow-up, respectively. The remaining 31,735 patients were included in the final analysis. Of these, 4318 (13.6%) patients received crystalloid fluid resuscitation during transfer to hospitals, while 27,417 (86.3%) patients did not. By using 1:1 PSM, the patients were 3024 in both fluid and non-fluid groups (Fig. 1).

Table 1 demonstrates the baseline characteristics, clinical manifestations, and outcomes between fluid and without fluid resuscitation groups. The patients in the fluid group were significantly younger (37.6 vs. 49.1 in years) and female gender dominant (76.2% vs. 59.8%). Korea, Malaysia, and Vietnam were the top three countries with enrolled patients. The overall mortality rates of eligible cases among studied countries were similar (Supplementary Table S2). In Korea, most patients (98.4%) did not receive crystalloid fluid resuscitation in the ambulance, while most patients (51.2%) received fluid support on hospital arrival in Malaysia. In patients who were administered fluid, traffic events (70.3%) were the first mechanism of injury, followed by slip or fall (19.4%) and physical strike (5.4%). Conversely, in patients without fluid resuscitation, slip or fall was the major cause (38.4%) and had a slightly higher rate than traffic events (34.8%). Head injury, face injury, and upper and lower extremities were the leading locations of traumatic injury in both groups, ranging from approximately 24.2% to 43.4% in the fluid group and 23.9% to 32.8% in the non-fluid group. For diagnosis, fractures, open wounds, and superficial injuries were the top three diagnoses with no obvious difference between the two groups. The ISS in the fluid group was higher than that in the non-fluid group. In the fluid group, 13% of patients had major trauma, which was significantly higher than the 4.1% in the non-fluid group. A total of 276 (0.9%) patients presented shock at the scene and near half of them (48.2%) received fluid resuscitation on the ambulance.

**Table 1:** Demographics, clinical characteristics, and outcomes in patients with/without receiving prehospital fluid resuscitation before and after propensity score matching.

Variable	Data before matching				Data after matching			
	Valid *N*	Non-fluid (*n* = 27,417)	Fluid (*n* = 4318)	*p*	Valid *N*	Non-fluid (*n* = 3024)	Fluid (*n* = 3024)	*p*
Age (years old)	31,735	49.1 ± 22.7	37.6 ± 19.7	< 0.001	6048	36.1 ± 19.1	36.2 ± 18.5	0.873
Sex (males)	31,735	16,394 (59.8)	3289 (76.2)	< 0.001	6048	2322 (76.8)	2329 (77.0)	0.831
**Country**	31,735			< 0.001	6048			0.917
Korea		24,060 (87.8)	396 (9.2)			385 (12.7)	396 (13.1)	
Malaysia		2900 (10.6)	3043 (70.5)			2434 (80.5)	2426 (80.2)	
Vietnam		440 (1.6)	232 (5.4)			188 (6.2)	182 (6.0)	
Others		17 (0.1)	647 (15.0)			17 (0.6)	20 (0.7)	
**Mechanism of injury**	31,735			< 0.001	6,048			0.982
Traffic events		9553 (34.8)	3036 (70.3)			2357 (77.9)	2344 (77.5)	
Slip or fall		10,522 (38.4)	837 (19.4)			417 (13.8)	426 (14.1)	
Physical strike		4069 (14.8)	232 (5.4)			138 (4.6)	141 (4.7)	
Others		3273 (11.9)	213 (4.9)			112 (3.7)	113 (3.7)	
**Location of injury**								
Head	31,735	8649 (31.5)	1623 (37.6)	< 0.001	6048	970 (32.1)	992 (32.8)	0.546
Face	31,735	8670 (31.6)	1043 (24.2)	< 0.001	6048	623 (20.6)	639 (21.1)	0.613
Neck	31,735	2043 (7.5)	238 (5.5)	< 0.001	6048	144 (4.8)	151 (5.0)	0.676
Thorax	31,735	2868 (10.5)	633 (14.7)	< 0.001	6048	394 (13.0)	409 (13.5)	0.570
Abdomen and pelvic	31,735	1279 (4.7)	384 (8.9)	< 0.001	6048	193 (6.4)	206 (6.8)	0.501
Spine	31,735	2395 (8.7)	208 (4.8)	< 0.001	6048	107 (3.5)	119 (3.9)	0.416
Upper extremity	31,735	6559 (23.9)	1456 (33.7)	< 0.001	6048	1078 (35.6)	1033 (34.2)	0.225
Lower extremity	31,735	8994 (32.8)	1873 (43.4)	< 0.001	6048	1394 (46.1)	1401 (46.3)	0.857
Skin	31,735	711 (2.6)	763 (17.7)	< 0.001	6048	514 (17.0)	528 (17.5)	0.634
Others	31,735	254 (0.9)	27 (0.6)	0.0496	6048	18 (0.6)	16 (0.5)	0.731
**Diagnosis of injury**								
Fracture	31,735	8781 (32.0)	1791 (41.5)	< 0.001	6048	1191 (39.4)	1205 (39.8)	0.713
Sprain, strain or dislocation	31,735	4668 (17.0)	318 (7.4)	< 0.001	6048	233 (7.7)	223 (7.4)	0.626
Cuts, bites or open wound	31,735	8448 (30.8)	1,327 (30.7)	0.914	6048	847 (28.0)	864 (28.6)	0.627
Bruise or superficial injury	31,735	10,102 (36.8)	2,122 (49.1)	< 0.001	6048	1452 (48.0)	1,462 (48.3)	0.797
Concussion	31,735	2908 (10.6)	567 (13.1)	< 0.001	6048	429 (14.2)	416 (13.8)	0.630
Organ system injury	31,735	1392 (5.1)	411 (9.5)	< 0.001	6048	196 (6.5)	200 (6.6)	0.835
Others	31,735	1608 (5.9)	134 (3.1)	< 0.001	6048	104 (3.4)	102 (3.4)	0.887
Injury severity score (ISS)	31,735	4.1 ± 5.1	6.9 ± 8.1	< 0.001	6048	6.4 ± 7.2	6.5 ± 7.4	0.501
Major trauma (ISS ≥ 16)	31,735	1,128 (4.1)	560 (13.0)	< 0.001	6048	335 (11.1)	347 (11.5)	0.626
Shock	31,735	143 (0.5)	133 (3.1)	< 0.001	6048	54 (1.8)	55 (1.8)	0.923
**Vital signs**								
SBP (mm-Hg)	31,735	133 ± 22	127 ± 24	< 0.001	6048	130 ± 22	129 ± 23	0.616
DBP (mm-Hg)	31,735	82.3 ± 15.0	74.2 ± 17.7	< 0.001	6048	76.9 ± 15.2	76.6 ± 15.9	0.488
HR (beats/min)	31,735	85.3 ± 15.8	85.4 ± 25.3	0.722	6048	87.1 ± 19.0	86.3 ± 21.0	0.094
RR (breaths/min)	31,735	17.5 ± 3.2	18.2 ± 4.9	< 0.001	6048	18.5 ± 3.7	18.3 ± 4.0	0.063
SpO_2_ (%)	31,735	97.7 ± 6.3	92.6 ± 21.6	< 0.001	6048	96.0 ± 14.1	95.2 ± 16.5	0.040
Total prehospital time (min)	7126	35 [26, 48]	38 [30, 50]	< 0.001	4735	36 [28, 47]	39 [30, 50]	< 0.001
Transport time (min)	8464	10 [7, 18]	10 [7, 15]	0.276	4772	10 [7, 15]	11 [7, 17]	0.006
ED stays (min)	29,636	175 [102, 305]	182 [91, 349]	0.856	5306	187 [114, 309]	204 [119,364]	< 0.001
Survived to discharge	31,735	27,770 (99.5)	4194 (97.1)	< 0.001	6048	2.992 (98.9)	2960 (97.9)	0.001
**Neurologic performance at discharge**				< 0.001				< 0.001
No symptoms at all	31,735	5450 (19.9)	572 (13.2)		6048	299 (9.9)	227 (7.5)	
Mild or slight disability	31,735	18,191 (66.3)	2539 (58.8)		6048	2118 (70.0)	1983 (65.6)	
Moderate disability	31,735	1927 (7.0)	485 (11.2)		6048	321 (10.6)	351 (11.6)	
Severe disability	31,735	1702 (6.2)	598 (13.8)		6048	254 (8.4)	399 (13.2)	
Deaths	31,735	147 (0.5)	124 (2.9)		6048	32 (1.1)	64 (2.1)	

*DBP* diastolic blood pressure, *ED* emergency department, *HR* heart rate, *ISS* injury severity score, *RR*  respiratory rate, *SBP*  systolic blood pressure, *SD*  standard deviation.

Data were presented as frequency (percentage) or mean ± standard deviation or median [Quartile 1, Quartile 3].

After 1:1 PSM, there were 3024 patients in both groups. No significant difference between groups was found (*p* < 0.05) in age, sex, mechanism, location, diagnosis, ISS, major trauma, and vital signs on the ambulance. The median total prehospital time was 39 min in the fluid group and 36 min in the non-fluid group. The total median time on ambulance transportation was 11 min in the fluid group and 10 min in the non-fluid group.

## Main results

### In-hospital mortality

In total, 96 (1.6%) patients died during hospitalization. The patients in the fluid group had a higher rate of in-hospital mortality than those in the non-fluid group (2.1% vs. 1.1%). The association between in-hospital mortality and transfusion of crystalloid solution is depicted in Table 2.

**Table 2:** The association between prehospital fluid resuscitation and outcomes in the original cohort, propensity-matched cohort, and IPTW cohort.

	Prehospital fluid resuscitation on the ambulance			
	Yes (n = 4318)	No (n = 27,417)	aOR (95% CI)*	*p*
Outcome of the original cohort (n = 31,735)				
**In-hospital mortality**				
Yes, n (%)	124 (2.9)	147 (0.54)	2.89 (1.92–4.34)	< 0.001
No, n (%)	4194 (97.1)	27,270 (99.46)		
**Functional outcome**				
Poor, n (%)	722 (16.7)	1849 (6.7)	1.92 (1.64–2.25)	< 0.001
Favoriable, n (%)	3596 (83.3)	25,568 (93.3)		

Prehospital fluid resuscitation on the ambulance				
	Yes (n = 3024))	No (n = 3024)	OR (95% CI)	*p*
***Outcome of the propensity-matched cohort (n = 6048)***				
**In-hospital mortality**				
Yes, n (%)	64 (2.1)	32 (1.1)	2.02 (1.32–3.10)	0.001
No, n (%)	2960 (97.9)	2992 (98.9)		
**Functional outcome**				
Poor, *n* (%)	463 (15.3)	286 (9.5)	1.73 (1.48–2.03)	< 0.001
Favoriable, *n* (%)	2561 (84.7)	2738 (90.5)		

	Prehospital fluid resuscitation on the ambulance			
	Yes (n = 8213.2)	No (n = 30,777.7)	OR (95% CI)	*p*
***Outcome of the IPTW cohort (n = 38,990.9)***^***#***^				
**In-hospital mortality**				
Yes, %	2.5	0.6	4.26 (3.50–5.20)	< 0.001
No, %	97.5	99.4		
**Functional outcome**				
Poor, %	15.2	7.1	2.33 (2.16–2.51)	< 0.001
Favorable, %	84.8	92.9		

*IPTW* inverse probability of treatment weighting, *CI*  confidence interval, *OR*  odds ratio, *aOR* adjusted odds ratio.

*Adjusted for age, sex, country, mechanism of injury, location of injury, diagnosis of injury, injury severity score, major trauma, shock, SBP, DBP, HR, and RR on the ambulance.

^#^The extreme weights were truncated at the 97th percentile.

**Figure 2:**
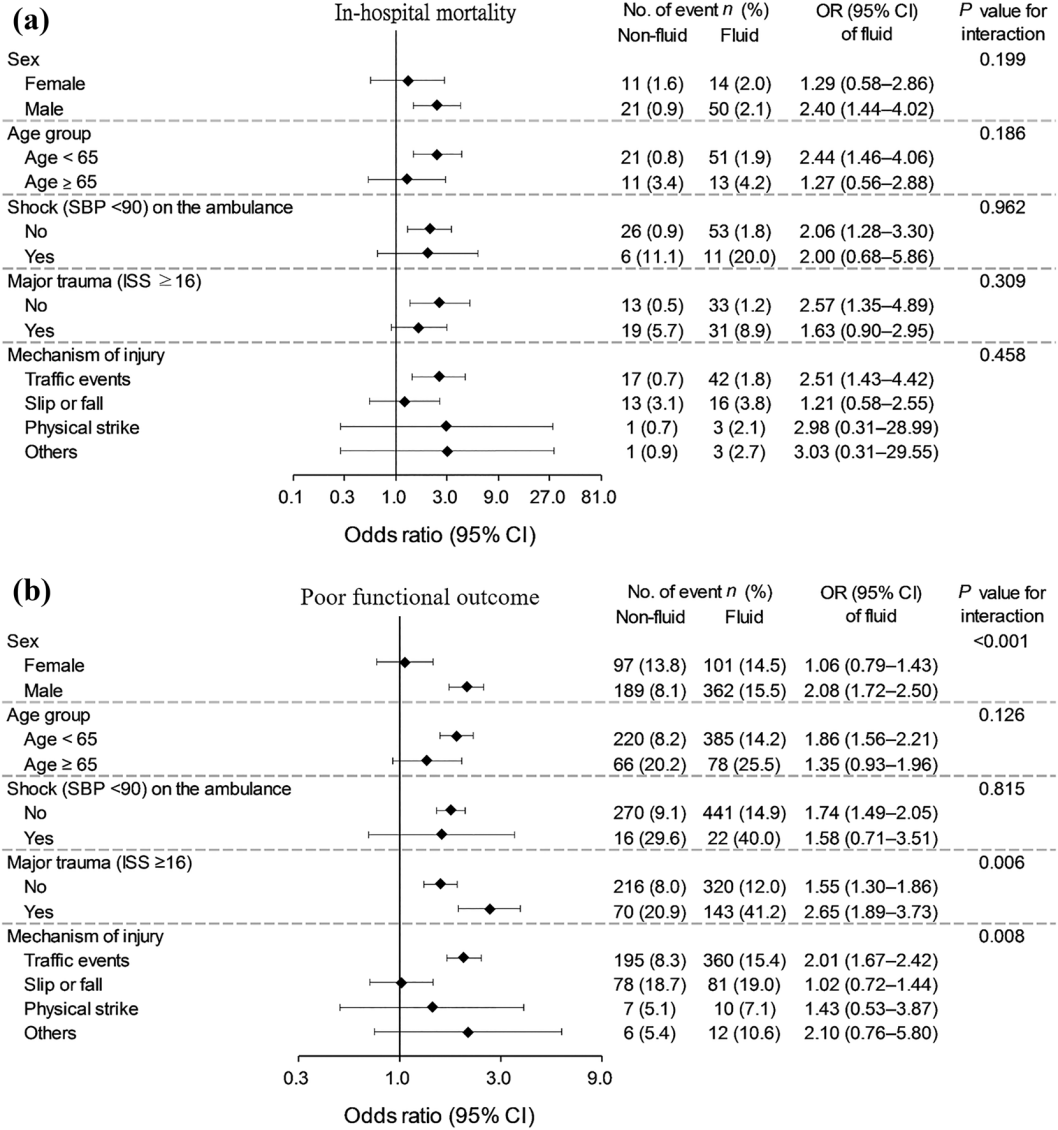
**(a)** Subgroup analysis for association between prehospital fluid resuscitation and in-hospital mortality, **(b)** subgroup analysis for association between prehospital fluid resuscitation and poor functional outcome. Figures are made by Microsoft PowerPoint 2019 and SPSS version 26.

In the PSM cohort, the patients who received fluid resuscitation during ambulance transport to hospitals had twice the risk for in-hospital mortality (crude odds ratio [OR] = 2.02, 95% confidence interval [CI] = 1.32. 3.10, *p* = 0.001). In the confounder-adjusted multivariate logistic regression analysis of the whole cohort, 124 (2.9%) patients had in-hospital mortality. After adjusting for age, sex, country, injury mechanism, location of injury, ISS, major trauma, and hypotension on the ambulance, the risk for in-hospital mortality of patients with fluid transfusion on the ambulance was higher than those without fluid transfusion (OR = 2.89, 95% CI = 1.92–4.34, *p* = 0.001). In the IPTW adjusted cohort, fluid transfusion on the ambulance was also associated with a higher risk of in-hospital mortality (OR = 4.26, 95% CI = 3.50–5.20, p < 0.001). Additionally, in the subgroup analysis of the effect of fluid transfusion, there was no significant interaction relationship in each subgroup including sex, age group, injury mechanism, hypotension on the ambulance, or major trauma (Fig. 2A).

### Poor functional outcome

At hospital discharge, 463 (15.3%) patients with prehospital fluid resuscitation on the ambulance had poor functional outcomes. On the other hand, 286 (9.5%) patients without any fluid on the ambulance had poor functional outcomes (Table 2). Traumatic patients who received prehospital fluid resuscitation on the ambulance had a higher risk of poor functional outcome (OR = 1.73, 95% CI = 1.48–2.03, *p* < 0.001) in the PSM cohort. On the other hand, in the entire cohort, 722 (16.7%) patients who received fluid resuscitation had poor functional outcomes. The confounders were well adjusted and controlled, and the association between poor functional outcome and prehospital fluid resuscitation were compared. Patients with prehospital fluid resuscitation on ambulance had a higher risk of poor functional outcome than those without prehospital fluid resuscitation (OR = 1.92, 95% CI = 1.64–2.25, *p* < 0.001). In the IPTW adjusted cohort, fluid transfusion on the ambulance was also associated with a higher risk of poor functional outcome (OR = 2.33, 95% CI = 2.16–2.51, *p* < 0.001). The subgroup analysis, displayed in Fig. 2B, indicated that male traumatic patients had a higher risk of poor functional outcome than female traumatic patients (OR = 2.08, 95% CI = 1.72–2.50, *p*_*interaction*_ < 0.001) if they received prehospital fluid resuscitation on arrival to hospitals. Furthermore, the effect of poor functional outcome of prehospital fluid resuscitation in patients with major trauma was more significant than that in patients without major trauma. Poor functional outcome was significantly noted in patients who received fluid resuscitation and were involved in traffic events as compared with other mechanisms of injury (OR = 2.01, 95% CI = 1.67. 2.42, *p*_*interaction*_ = 0.008).

## Discussion

In this cross-national, multi-center, large-scale retrospective cohort study of the populations in Asian countries, we found that prehospital crystalloid fluid resuscitation was associated with increased in-hospital mortality and poor functional outcome in traumatic patients. The results were consistent in the PSM propensity score-matched and the original cohorts. Furthermore, we conducted inverse probability of treatment weighting (IPTW) with stabilized weight based on propensity score to increase the statistical power. All the methods reach the same direction and comparable results (Table 2). The risk of fluid transfusion was approximately twice as high in both in-hospital mortality and poor functional outcome. The administration of fluid transfusion before arrival to hospitals in the male sex, major trauma, and traffic event population were more vulnerable to poor functional outcomes. To the best of our knowledge, this is the first study to adopt a cross-national, multicenter traumatic database to investigate the association of outcome and fluid resuscitation in Asian countries. The sample size was large, and the explanatory power on the results was sufficient and convincing after adjusting for confounders.

In this study, the population included all patients who suffered a traumatic injury and were brought to the hospitals by the initiating EMS. Approximately 5.3% (1,688/31,735) experienced major trauma (ISS > 15). This rate was similar to that in other regions. The Dutch National Trauma Registry (DNTR) was nationally coordinated to 11 trauma regions. Annually, among approximately 80,000 included participants, approximately 5% are considered to suffer major trauma^18^. Additionally, in our study, in-hospital mortality accounted for 1.6% of the propensity-matched cohort. However, the overall trauma mortality rate varied depending on age distribution, injury type, and injury severity.

Our results are consistent with those proposed by Haut et al. in a large American NTDB^4^. The NTDB is the largest collection of trauma registry data ever assembled, permitting the examination of data from trauma patients. However, the NTDB recruited only Americans and did not report prehospital transport times. In the NTDB study, the authors failed to control for transport time within the multiple regression model or perform a stratified analysis in traumatic patients. In the current study, we had the strength to match the prehospital time intervals in the analysis of the effect of prehospital fluid resuscitation. The current study extended the population from multiple countries and provided evidence for Asian populations. We enrolled 31,735 Asian patients from 8 different countries in Asia. The results demonstrated an almost two-fold higher risk for both in-hospital mortality and poor functional outcome in both the PSM and original cohorts by multivariate-adjusted logistic regression. Whereas, Haut et al. performed an impressive retrospective cohort study of 776,734 patients from the NTDB using records from more than 600 US trauma centers^4^. The harm associated with prehospital IV placement is significant for all trauma victims (OR = 1.11, 95% CI = 1.06–1.17). This phenomenon was significantly stressed in hypotensive patients. The negative association between prehospital fluid transfusion and death was more exaggerated (OR = 1.44, 95% CI = 1.29–1.59). Similar to Haut’s result, patients with major trauma (ISS ≥ 16) were more vulnerable to poor outcomes if they received fluid resuscitation on the ambulance. Notably, the definition of “IV fluid” in our study was the patients who simply received intravenous fluid rather than the either “IV fluid” or “on IV catheter” in Haut’s report, which raised a critical discussion on a confusion of the population. Some patients who were classified into the fluid group in Haut’s study did not receive prehospital fluid resuscitation; however, they only received IV catheter deployment^19–21^.

 One theory implies a delay in transport to hospitals and receiving definitive care. The placement of the intravenous route is not only associated with increased time at scene but also increased overall time to the hospital. In some cases, the time to place an IV exceeds that of the actual transport itself. Smith et al. recruited 52 trauma cases and found that all cases cost more time for intravenous establishment. The authors concluded that infused fluid volume had little influence on final outcomes^22^. Another theory regarding the potential disadvantage of fluid resuscitation in the prehospital stage is based on the concept of “popping the clot.” The theory of permissive hypotension emphasizes limited fluids during the early stages of treatment for hemorrhagic status^23^. This suggests that in patients who have stopped bleeding temporarily from vasoconstriction and hypotension, intravenous fluids may increase systolic blood pressure and cause patients to re-bleed if the bleeding source was not yet definitely controlled. Bickell et al. demonstrated the survival benefits of delayed aggressive fluid resuscitation until definite surgery in a 598-adults penetrating torso injuries study^24^. However, due to the well-matched cohort in the analysis, our results did not demonstrate an interaction between the administration of crystalloid fluid transfusion and hypotensive shock (< 90 mmHg) in the subgroup analysis (Fig. 2).

Conversely, some studies favor fluid resuscitation in the prehospital stage. Hampton et al. analyzed the effect of prehospital fluid resuscitation in 1,245 trauma patients enrolled in the Prospective Observational Multi-center Massive Transfusion Study (PROMMTT)^25^, and the results revealed that pre-hospital fluid administration was associated with decreased in-hospital mortality (hazard ratio = 0.84, 95% CI = 0.72–0.98, *p* = 0.03)^26^. However, one of the potential problems that may influence the results originated from data that were missing 44% of the data regarding on-ambulance systolic blood pressure because pre-hospital data collection was not the priority of the PROMMTT study. Another reason is that the patients who needed prehospital resuscitation in the PROMMTT study received mixed pure saline and blood products. The patients in the fluid group received a median pre-hospital fluid volume of 700 mL, which was less than the 1–2 L recommended by the current guideline. The effect of dilutional coagulopathy and hyperchloremic metabolic acidosis may tremendously decrease, which is different from our results. Our patients received only crystalloids during prehospital fluid resuscitation. Accordingly, further randomized controlled trials are required.

Dextrose may be contraindicated in resuscitation. In this study, we found that crystalloid fluids used included normal saline, dextrose solution, and Hartmann's solution. Very few patients in the current study received dextrose solution (12 patients, 0.04%). One reason may be that the patient suffered from complications such as hypoglycemia, but this was not documented in the database. The actual reason for dextrose solution administration was not obtained.

There were some limitations to the current study. First, it was limited by the nature of the retrospective cohort studies. Missing data could not be avoided in a cross-national, multicenter, traumatic database registry. We assumed that the data were missed randomly and did not influence the direction of the observed associations. In our study cohort, the exclusion rate was approximately 33% due to missing variables (Fig. 1). The exclusion protocol used in this study was strict. No imputation or other methods were applied to the missing variables. Second, the amount of fluid in the current study was difficult to quantize, even though most patients received one bag of normal saline (500 mL) by records. We are not sure whether a bag of normal saline was totally infused to patients or just a few millimeters, but it was documented as one bag. Third, although PATOS used a predefined record form, differences existed between hospitals, healthcare delivery systems, and countries. For example, the impact of the level of hospitals to which the patients were transferred could be a concern. The quality of post-trauma center arrival trauma care may significantly influence the outcomes. In addition, road traffic laws and conditions differ between countries. The transport policy has not been fully explored. Fourth, selection bias may still exist although the cases were from a large-scale, multi-center, and countries PATOS database. Patients enrolled in PATOS had to initiate the EMS system and were brought to hospitals by an ambulance. In an analysis of 103,029 patients between January 2010 and December 2012, patients who used private transportation had reduced in-hospital mortality compared with those who waited for an ambulance^27^. These patients by private transportation were not included in the current study. Also, most of the patients (80%) in the propensity-matched cohort were from Malaysia, which might raise the concern of a selection bias and might not representative for the whole Asia population. Fifth, PATOS database included AIS codes and summarized ISS rather than AIS for each patient. Although no separated AIS was available, AIS coding was performed by trained emergency physicians and nurses, with relatively stable coding quality. Furthermore, most EMS in Asia are not physician-staffed, unlike what is widely seen in Europe. The practice of the Asian EMSs is to use paramedics on the scene. Information on the level of the emergency medical technicians participating in every event was unavailable. Since the emergency medical technician can perform basic intravenous catheterization, the participating EMT had few effects on the current study. Since the basic emergency medical technician is able to perform intravenous catheterization, the level of technician affects little in the current study. Sixth, the PATOS database did not include the short-term outcome such as 24 h mortality. The causal link between limited volume resuscitation and hospital mortality might be difficult to establish, and the application of the current results should be conservative. Seventh, since in our studied population the critically sick patients were in low numbers and most patients would probably not have required a fluid administration, the positivity assumption with regard to fluid resuscitation may raise a concern and the results should be cautiously interpreted when being applied in different EMS configurations. Lastly, we did not mention intra-hospital treatment in the current study. Intra-hospital treatment, including blood transfusion in the ED and time taken to receive definite treatment, such as angiography and transarterial embolization, may affect the outcome. Quality care during hospitalization affects the complication rate, thereby causing a potentially poor outcome. Therefore, these factors will be analyzed in future studies.

In patients with trauma, we found that prehospital fluid resuscitation with crystalloids may be associated with higher in-hospital mortality and poor functional outcome at hospital discharge in the Asian countries studied. In the subgroup characterized by young, males, non-hypotension, non-major trauma, and traffic events, the adverse effect of prehospital fluid resuscitation on outcomes is more significant than that of the old, female, major trauma, and other trauma events, respectively (Supplementary Information).

## Supplementary Information

Supplementary Table S1.

Supplementary Table S2.

## References

[1] Dadoo S (2017). Prehospital fluid administration in trauma patients: A survey of state protocols. Prehosp. Emerg. Care.

[2] Kauvar DS, Lefering R, Wade CE (2006). Impact of hemorrhage on trauma outcome: An overview of epidemiology, clinical presentations, and therapeutic considerations. J. Trauma.

[3] de Crescenzo C, Gorouhi F, Salcedo ES, Galante JM (2017). Prehospital hypertonic fluid resuscitation for trauma patients: A systematic review and meta-analysis. J. Trauma Acute Care Surg..

[4] Haut ER (2011). Prehospital intravenous fluid administration is associated with higher mortality in trauma patients: A National Trauma Data Bank analysis. Ann. Surg..

[5] Ley EJ (2011). Emergency department crystalloid resuscitation of 1.5 L or more is associated with increased mortality in elderly and nonelderly trauma patients. J. Trauma.

[6] Heuer M (2015). Prehospital fluid management of abdominal organ trauma patients–A matched pair analysis. Langenbecks Arch. Surg..

[7] Schreiber MA (2015). A controlled resuscitation strategy is feasible and safe in hypotensive trauma patients: results of a prospective randomized pilot trial. J. Trauma Acute Care Surg..

[8] Geeraedts Jr LM, Pothof LA, Caldwell E, de Lange-de Klerk ES, D’Amours (2015). Prehospital fluid resuscitation in hypotensive trauma patients: Do we need a tailored approach?. Injury.

[9] Kwan I, Bunn F, Chinnock P, Roberts I (2014). Timing and volume of fluid administration for patients with bleeding. Cochrane Database Syst. Rev..

[10] Revell M, Porter K, Greaves I (2002). Fluid resuscitation in prehospital trauma care: A consensus view. Emerg. Med. J..

[11] Chen CH (2020). Association between prehospital time and outcome of trauma patients in 4 Asian countries: A cross-national, multicenter cohort study. PLoS Med.

[12] Kong SY (2018). Pan-Asian Trauma Outcomes Study (PATOS): Rationale and methodology of an International and Multicenter Trauma Registry. Prehosp. Emerg. Care.

[13] von Elm E (2007). The Strengthening the Reporting of Observational Studies in Epidemiology (STROBE) statement: guidelines for reporting observational studies. PLoS Med..

[14] Banks JL, Marotta CA (2007). Outcomes validity and reliability of the modified Rankin scale: Implications for stroke clinical trials: A literature review and synthesis. Stroke.

[15] Rittenberger JC, Raina K, Holm MB, Kim YJ, Callaway CW (2011). Association between cerebral performance category, modified Rankin Scale, and discharge disposition after cardiac arrest. Resuscitation.

[16] Wilson JT (2002). Improving the assessment of outcomes in stroke: Use of a structured interview to assign grades on the modified Rankin Scale. Stroke.

[17] David FN, Johnson NL (1951). The effect of non-normality on the power function of the F-test in the analysis of variance. Biometrika.

[18] Van Ditshuizen JC (2021). The definition of major trauma using different revisions of the abbreviated injury scale. Scand. J. Trauma Resusc. Emerg. Med..

[19] Rivkind AI, Ganor O, Glassberg E (2014). Prehospital intravenous fluid administration is associated with higher mortality in trauma patients. Ann. Surg..

[20] Niven DJ, Stelfox HT, Ball CG, Kirkpatrick AW (2014). Prehospital intravenous fluid administration is associated with higher mortality in trauma patients: A National Trauma Data Bank analysis. Ann. Surg..

[21] Champion HR (2014). Prehospital intravenous fluid administration is associated with higher mortality in trauma patients. Ann. Surg..

[22] Smith JP, Bodai BI, Hill AS, Frey CF (1985). Prehospital stabilization of critically injured patients: A failed concept. J. Trauma.

[23] Carrick MM, Leonard J, Slone DS, Mains CW, Bar-Or D (2016). Hypotensive resuscitation among trauma patients. Biomed. Res. Int..

[24] Bickell WH (1994). Immediate versus delayed fluid resuscitation for hypotensive patients with penetrating torso injuries. N. Engl. J. Med..

[25] Holcomb JB (2013). The prospective, observational, multicenter, major trauma transfusion (PROMMTT) study: Comparative effectiveness of a time-varying treatment with competing risks. JAMA Surg..

[26] Hampton DA (2013). Prehospital intravenous fluid is associated with increased survival in trauma patients. J. Trauma Acute Care Surg..

[27] Wandling MW, Nathens AB, Shapiro MB, Haut ER (2018). Association of prehospital mode of transport with mortality in penetrating trauma: A trauma system-level assessment of private vehicle transportation vs ground emergency medical services. JAMA Surg..

