# Supplementary material for: Association between prehospital fluid resuscitation with crystalloids and outcome of trauma patients in Asia by a cross-national multicenter cohort study
Source: Sci Rep. 2022 Mar 8;12:4100. doi: 10.1038/s41598-022-06933-x (PMC8902907; doi:10.1038/s41598-022-06933-x)
Supplement: Supplementary file 1 — Supplementary Table S1. [file 41598_2022_6933_MOESM1_ESM.pdf]

STROBE Statement—checklist of items that should be included in reports of observational studies

	Item No.	Recommendation	Page No.	Relevant text from manuscript
**Title and abstract**	1	(*a*) Indicate the study's design with a commonly used term in the title or the abstract	1	Association between prehospital fluid ……
		(*b*) Provide in the abstract an informative and balanced summary of what was done and what was found	2	Prehospital fluid resuscitation with……
**Introduction**				
Background/rationale	2	Explain the scientific background and rationale for the investigation being reported	3-4	Traumatic injury is a universal problem……
Objectives	3	State specific objectives, including any prespecified hypotheses	4	Trauma patients generally include……
**Methods**				
Study design	4	Present key elements of study design early in the paper	5	This cross-national, multicentre……
Setting	5	Describe the setting, locations, and relevant dates, including periods of recruitment, exposure, follow-up, and data collection	5	The PATOS coordination center……
Participants	6	(*a*) *Cohort study*—Give the eligibility criteria, and the sources and methods of selection of participants. Describe methods of follow-up<br>*Case-control study*—Give the eligibility criteria, and the sources and methods of case ascertainment and control selection. Give the rationale for the choice of cases and controls<br>*Cross-sectional study*—Give the eligibility criteria, and the sources and methods of selection of participants	6	We included patients aged > 18 years……
		(*b*) *Cohort study*—For matched studies, give matching criteria and number of exposed and unexposed<br>*Case-control study*—For matched studies, give matching criteria and the number of controls per case	6	We included patients aged > 18 years……
Variables	7	Clearly define all outcomes, exposures, predictors, potential confounders, and effect modifiers. Give diagnostic criteria, if applicable	6-7	We included the variables……

Data sources/ measurement	8*	For each variable of interest, give sources of data and details of methods of assessment (measurement). Describe comparability of assessment methods if there is more than one group	*5*	The PATOS coordination center provides……
Bias	9	Describe any efforts to address potential sources of bias	5	Some of the participating hospitals…
Study size	10	Explain how the study size was arrived at	NA	NA

Continued on next page

Quantitative variables	11	Explain how quantitative variables were handled in the analyses. If applicable, describe which groupings were chosen and why	7	The primary outcome……
Statistical methods	12	(*a*) Describe all statistical methods, including those used to control for confounding	7-9	Data collection and processing were performed by…
		(*b*) Describe any methods used to examine subgroups and interactions	8	To avoid potential unbalanced demographics……
		(*c*) Explain how missing data were addressed	8	To avoid potential unbalanced demographics……
		(*d*) *Cohort study*—If applicable, explain how loss to follow-up was addressed<br>*Case-control study*—If applicable, explain how matching of cases and controls was addressed<br>*Cross-sectional study*—If applicable, describe analytical methods taking account of sampling strategy	8	Each patient in the fluid……
		(*e*) Describe any sensitivity analyses	8-9	Each patient in the fluid……
**Results**				
Participants	13*	(a) Report numbers of individuals at each stage of study—eg numbers potentially eligible, examined for eligibility, confirmed eligible, included in the study, completing follow-up, and analysed	10	We preliminary enrolled 47,617 trauma…
		(b) Give reasons for non-participation at each stage	NA	NA
		(c) Consider use of a flow diagram	Figure 1	Figure 1
Descriptive data	14*	(a) Give characteristics of study participants (eg demographic, clinical, social) and information on exposures and potential confounders	10-11	Table 1 demonstrates the baseline……
		(b) Indicate number of participants with missing data for each variable of interest	NA	NA
		(c) *Cohort study*—Summarise follow-up time (eg, average and total amount)	10-11	Table 1 demonstrates the baseline……
Outcome data	15*	*Cohort study*—Report numbers of outcome events or summary measures over time	11	In total, 96 (1.6%) patients died during……
		Case-control study—Report numbers in each exposure category, or summary measures of exposure		
		Cross-sectional study—Report numbers of outcome events or summary measures		
Main results	16	(*a*) Give unadjusted estimates and, if applicable, confounder-adjusted estimates and their precision (eg, 95% confidence interval). Make clear which confounders were adjusted for and why they were included	11-12	In the PSM cohort, the patients……
		(*b*) Report category boundaries when continuous variables were categorized	11-12	In the PSM cohort, the patients……

	(*c*) If relevant, consider translating estimates of relative risk into absolute risk for a meaningful time period	NA	NA

Continued on next page

Other analyses	17	Report other analyses done—eg analyses of subgroups and interactions, and sensitivity analyses	NA	NA
**Discussion**				
Key results	18	Summarise key results with reference to study objectives	13	In this cross-national, multi-center, large-scale……
Limitations	19	Discuss limitations of the study, taking into account sources of potential bias or imprecision. Discuss both direction and magnitude of any potential bias	16-17	There were some limitations to the current……
Interpretation	20	Give a cautious overall interpretation of results considering objectives, limitations, multiplicity of analyses, results from similar studies, and other relevant evidence	13-16	In this study, the population included……
Generalisability	21	Discuss the generalisability (external validity) of the study results	13-16	In this study, the population included……
**Other information**				
Funding	22	Give the source of funding and the role of the funders for the present study and, if applicable, for the original study on which the present article is based	22	This study was funded by the Taiwan Ministry……

*Give information separately for cases and controls in case-control studies and, if applicable, for exposed and unexposed groups in cohort and cross-sectional studies.

Note: An Explanation and Elaboration article discusses each checklist item and gives methodological background and published examples of transparent reporting. The STROBE checklist is best used in conjunction with this article (freely available on the Web sites of PLoS Medicine at http://www.plosmedicine.org/, Annals of Internal Medicine at http://www.annals.org/, and Epidemiology at http://www.epidem.com/). Information on the STROBE Initiative is available at www.strobe-statement.org.
