# Supplementary material for: Association between prehospital fluid resuscitation with crystalloids and outcome of trauma patients in Asia by a cross-national multicenter cohort study
Source: Sci Rep. 2022 Mar 8;12:4100. doi: 10.1038/s41598-022-06933-x (PMC8902907; doi:10.1038/s41598-022-06933-x)
Supplement: Supplementary file 2 — Supplementary Table S2. [file 41598_2022_6933_MOESM2_ESM.docx]

| Supplementary table 2. Comparison of mortality between sites | | | | | | |
| --- | --- | --- | --- | --- | --- | --- |
| Country | Total | Korea | Malaysia | Vietnam | Others | *p* |
| mortality, n (%) | 271 (0.86) | 200 (0.82) | 52 (0.88) | 5 (0.81) | 6(0.87) | 0.843 |
